# GIFTed Demons: deformable image registration with local structure-preserving regularization using supervoxels for liver applications

**DOI:** 10.1117/1.JMI.5.2.024001

**Published:** 2018-04-04

**Authors:** Bartłomiej W. Papież, James M. Franklin, Mattias P. Heinrich, Fergus V. Gleeson, Michael Brady, Julia A. Schnabel

**Affiliations:** a University of Oxford, Institute of Biomedical Engineering, Department of Engineering Science, Oxford, United Kingdom; b University of Oxford, Department of Oncology, Oxford, United Kingdom; c University of Lübeck, Institute of Medical Informatics, Lübeck, Germany; d Oxford University Hospitals NHS Trust, Churchill Hospital, Department of Radiology, Oxford, United Kingdom; e King’s College London, School of Biomedical Engineering and Imaging Sciences, London, United Kingdom

**Keywords:** deformable image registration, adaptive regularization, guided image filtering, supervoxels

## Abstract

Deformable image registration, a key component of motion correction in medical imaging, needs to be efficient and provides plausible spatial transformations that reliably approximate biological aspects of complex human organ motion. Standard approaches, such as Demons registration, mostly use Gaussian regularization for organ motion, which, though computationally efficient, rule out their application to intrinsically more complex organ motions, such as sliding interfaces. We propose regularization of motion based on supervoxels, which provides an integrated discontinuity preserving prior for motions, such as sliding. More precisely, we replace Gaussian smoothing by fast, structure-preserving, guided filtering to provide efficient, locally adaptive regularization of the estimated displacement field. We illustrate the approach by applying it to estimate sliding motions at lung and liver interfaces on challenging four-dimensional computed tomography (CT) and dynamic contrast-enhanced magnetic resonance imaging datasets. The results show that guided filter-based regularization improves the accuracy of lung and liver motion correction as compared to Gaussian smoothing. Furthermore, our framework achieves state-of-the-art results on a publicly available CT liver dataset.

## 1 Introduction

Deformable image registration (DIR) of medical image volumes is an essential component of many biomedical image analysis applications.^1^ For example, computed tomography (CT) volumes are typically acquired several times over the course of radiotherapy, and DIR can help monitor changes to anatomy and pathology.^2^ Similarly, DIR can improve the quality assessment of dose delivery during radiotherapy.^3^^,^^4^ Furthermore, DIR can also increase the efficiency of CT data annotation by propagating an expert’s annotation from one volume (e.g., radiotherapy planning or an atlas)^5^ to follow-up image volumes. Likewise, quantitative analysis of temporal functional imaging, e.g., dynamic contrast-enhanced magnetic resonance imaging (DCE-MRI), is often challenged by the substantial motions among consecutive scans, in particular sliding motions at the lung and liver interface.^6^ Estimation of plausible organ motion is an ill-posed, high-dimensional, numerical optimization problem typically associated with millions of possible solutions.^7^ It follows that the transformations computed by an algorithm depend upon the chosen regularization model, and so DIR for medical applications remains a challenging task.^8^

### 1.1 Related Work

A convolution-based regularization model for DIR was first reported by Thirion,^9^ in which the so-called “Demons” registration algorithm was introduced. In the Demons registration algorithm, optimization is decomposed into two alternating subproblems: minimizing the similarity measure and followed by regularization of the displacement field by Gaussian smoothing of the currently estimated displacement field.^10^ However, such an intrinsically isotropic motion (regularization) model is inadequate for complex thoracic or abdominal motions, where locally discontinuous motions (sliding) are typical.

To address this limitation, a number of alternative regularization methods have been reported. For example, in Ref. 11, locally adaptive regularization was proposed to “shape” the Gaussian kernels according to a local stiffness parameter. Similarly, adaptive anisotropic filtering^12^^,^^13^ and local affine adaptive regularization^14^ have been proposed. However, each of these approaches yields deformations that smooth over sliding interfaces. Other developments of Demons registration applied to specific biomedical problems include constraining the transformation to be one-to-one, differentiable, that is to say a diffeomorphism.^15^ Filtering the displacement field was also proposed in Ref. 16, where local averaging based on initial bone segmentation ensures a rigidity constraint appropriate to the application. Similarly, biologically relevant tissue characteristics for cardiac motion estimation led to the incompressible Demons (iLogDemons) using the Helmholtz decomposition of deformations.^17^ Aside from Demons registration, other approaches have been proposed for DIR that use locally adaptive regularization. These include, for example, a variational approach to image registration with locally varying regularization.^18^ A summary of joint flow- and image-driven anisotropic regularization models for variational image registration is presented in Ref. 19. While such methods report promising results, they produce smooth transformations that fundamentally set limits on their application to abdominal imaging.

More recently, DIR algorithms with discontinuity preserving properties at lung interfaces have been reported.^20^^–^^28^ However, such algorithms generally require prior knowledge about the locations of discontinuities in deformations to be included into the optimization process. Such prior information may be incorporated in the form of segmentation of sliding surfaces using either a “motion mask”^29^ or an automatically detected mask.^22^ Another approach to improve accuracy of DIR algorithms is to perform registration separately for lungs and other anatomical regions.^30^^,^^31^ Unlike the liver, automated lung segmentation from CT is relatively straightforward due, primarily, to the fact that there is a large difference in attenuation between normal lung parenchyma and the surrounding tissue.^32^ Liver motion is also far more complex than that of the lungs, as the liver exhibits not only sliding motions against thoracic cage but also moves against other surrounding organs in the abdomen and the abdominal wall.^33^ Furthermore, the low contrast of liver tissue in CT makes accurate (and fast) segmentation of sliding surfaces in the abdomen challenging,^34^ so a DIR method that does not require explicit segmentation of the liver surface remains an attractive option.

### 1.2 Contributions

In this work, we present an approach to regularization that implicitly incorporates prior knowledge about the medical volumes to be registered in a natural manner. Our method is related to Demons registration, but we replace Gaussian regularization by a guided image filtering technique,^35^ a method we call GIFTed Demons. Guided image filtering is able to incorporate additional (e.g., anatomical) information from so-called “guidance” images in a computationally efficient manner (unlike time-consuming bilateral filtering).^27^ For example, instead of performing an accurate liver segmentation to guide the discontinuities of deformation, in this paper, we use a more straightforward pseudosegmentation generated by performing “simple linear iterative clustering” (SLIC) algorithm.^36^ In our previous work,^37^ such a locally adaptive regularization model was based on the SLIC algorithm, which had been initialized multiple times in a random fashion. This enabled the propagation of discontinuities to the estimated displacement field corresponding to image edges and details, while at the same time preserving the smoothness of homogeneous areas. In this paper, we show in addition that locally adaptive regularization can also be achieved using several layers of SLICs with different sizes (volumes) simultaneously, leading to multiscale SLIC regularization that is able to model piecewise smooth motions. Both models perform locally adaptive regularization, which can effectively deal with highly complex motions. We have performed extensive quantitative evaluation of our method using two clinical four-dimensional (4-D) datasets consisting of: (i) a set of publicly available CT liver scans^24^ and (ii) in-house DCE-MRI liver sequences of patients with cancer.^37^ Our results demonstrate that our registration method significantly improves registration accuracy as compared to state-of-the-art isotropic diffusion registration. Furthermore, for the benchmark CT liver dataset,^24^ a DIR using our regularization model yields results that are competitive with the state-of-the-art. In summary, SLIC improves propagation of discontinuities in the estimated transformations (either via random multichannel image guidance or via multiscale image guidance). Our method offers promising results while avoiding computationally intensive and potentially error-prone prior segmentation to model sliding boundaries explicitly.

### 1.3 Overview

The registration model is presented in Sec. 2. Since the approach is based on a registration algorithm that uses isotropic regularization, this aspect is briefly presented in Sec. 2.1. In Sec. 2.2, we present a general convolution-based regularization model, and then Sec. 2.3 introduces guided filters and their application for displacement filtering. Section 2.4 discusses a number of modified guidance images generated using supervoxels, which can deal effectively with sliding motions and exploit the computationally attractive guided image filtering technique presented in Sec. 2.3. Section 4 presents results of using the datasets described in Sec. 3. These results are compared against state-of-the-art diffusion registration frameworks. Results showing our approach to accurately align liver volumes, while preserving naturally occurring sliding motions, are discussed in Sec. 5.

## 2 Methods

### 2.1 Deformable Image Registration with Isotropic Diffusion Regularization

In a typical approach to motion correction,^7^ DIR is applied between two three-dimensional (3-D) volumes [from a spatio-temporal (4-D) dataset] containing either an anatomical or a functional representation of a patient’s region of interest. The primary aim of DIR is to estimate a plausible transformation $\phi (\overset{\to }{x})=\overset{\to }{x}+\overset{\to }{u}(\overset{\to }{x})$ (which is defined with respect to a displacement field $\vec{u}$) that maps each point of a source image $I_{M}$ to the most similar point in the reference image $I_{F}$. Generally, DIR can be defined as an optimization problem, minimizing an energy function $E(\vec{u})$

$$\arg\;\underset{\overset{\to }{u}}{\min}[E(\overset{\to }{u})=E_{\mathrm{SIM}}(I_{F},I_{M},\overset{\to }{u})+\alpha E_{\mathrm{REG}}(\overset{\to }{u})],$$ (1)

where $E_{\mathrm{SIM}}$ estimates image data similarity and $E_{\mathrm{REG}}$ regularizes (the “plausibility” of) the estimated displacement field. The weighting parameter α balances the influence of similarity and regularization.

Minimization of the energy function $E(\vec{u})$ [given by Eq. (1)] can be solved via the corresponding Euler–Lagrange equation (see e.g., Ref. 7)

$$f_{\mathrm{SIM}}(I_{F},I_{M},\overset{\to }{u})-\alpha A_{\mathrm{REG}}(\overset{\to }{u})=\overset{\to }{0},$$ (2)

where $f_{\mathrm{SIM}}$ is the force term that corresponds to the similarity measure $E_{\mathrm{SIM}}$ and $A_{\mathrm{REG}}$ is the partial differential operator used to regularize the displacement field according to the regularization term $E_{\mathrm{REG}}$. In the simplest case, when a one-to-one intensity mapping among the input images can be assumed (e.g., for CTs), we can consider the symmetric Demons force $f_{\mathrm{SIM}}$ related to a normalized version of the sum of squared differences (SSD) (for details see Ref. 15)

$$f_{\mathrm{SIM}}[\overset{\to }{u}(\overset{\to }{x})]=\frac{I_{F}(\overset{\to }{x})-I_{M}[\overset{\to }{x}+\overset{\to }{u}(\overset{\to }{x})]}{{\left\|\nabla I(\overset{\to }{x})\right\|}^{2}+\lambda \kappa^{2}(\overset{\to }{x})}\nabla I(\overset{\to }{x}),$$ (3)

where $\nabla I(\overset{\to }{x})=\frac{1}{2}\{\nabla I_{F}(\overset{\to }{x})+\nabla I_{M}[\overset{\to }{x}+\overset{\to }{u}(\overset{\to }{x})]\}$ is a symmetric gradient, $\kappa (\overset{\to }{x})=I_{F}(\overset{\to }{x})-I_{M}[\overset{\to }{x}+\overset{\to }{u}(\overset{\to }{x})]$ is a local estimate of noise, and λ is a weighting parameter that controls the maximum step length. Other similarity measures for multimodal registration can also be applied (see e.g., Ref. 38 for a local correlation coefficient or Ref. 39 for the correlation ratio and mutual information). A frequently chosen regularization term $E_{\mathrm{REG}}$ is isotropic diffusion (see e.g., Ref. 7)

$$E_{\mathrm{REG}}(\overset{\to }{u})=\frac{1}{2}\int_{\Omega }\overset{n}{\underset{j=1}{\sum }}{\left\|\nabla {\overset{\to }{u}}_{i}(\overset{\to }{x}))\right\|}^{2}d\overset{\to }{x},$$ (4)

with the corresponding partial differential operator

$$A_{\mathrm{REG}}[\overset{\to }{u}(\overset{\to }{x})]=-\Delta \overset{\to }{u}(\overset{\to }{x}),$$ (5)

where Δ denotes the Laplace operator. Diffusion regularization penalizes large gradients in the displacement field.^7^

### 2.2 Convolution-Based Regularization Model

Diffusion regularization [Eq. (4)] can be achieved by smoothing the displacement field $\vec{u}$ with an isotropic Gaussian kernel, as originally proposed by Thirion.^9^ Similarly, it has been suggested that the diffusion process exploits the fact that the Gaussian kernel is the Green’s function of the diffusion equation.^12^^,^^40^ It follows that the solution is approximated via iteratively repeated convolution with a Gaussian kernel G on the displacement field

$${\overset{\to }{u}}_{k+1}(\overset{\to }{x})=G*[{\overset{\to }{u}}_{k}(\overset{\to }{x})\circ f_{\mathrm{SIM}}(\overset{\to }{x})],$$ (6)

where ${\vec{u}}_{k}$ is the displacement field estimated at iteration k and ∘ is the composition operator.^15^

However, diffusion regularization is homogeneous and isotropic and, so, constrains the estimated deformations to be smooth, independent of location or direction. First, if the amount of regularization (given by the standard deviation of the Gaussian filter) is excessive, fine details of the displacement field are not well preserved. Second, if the standard deviation of the Gaussian filter is too small, the estimated deformation field is highly sensitive to image noise.^11^ Third, in the case of a sliding motion among objects, the smoothness constraint assumption is often violated at object boundaries resulting in inaccurate estimation of the deformation field close to such boundaries.^27^ Fortunately, the Gaussian kernel can be replaced by more powerful regularization, for example, nonstationary Gaussian kernels,^11^ anisotropic diffusion,^13^ bilateral filtering to counter occlusions,^40^ or to enable sliding motion,^27^ or locally adaptive image-driven curvature regularization^12^ to ensure physiologically more realistic deformations. In the following sections, we will show how to enforce the plausibility of the estimated displacement field, e.g., preservation of sliding motion between the liver and the lung boundaries while not requiring prior liver segmentation.

### 2.3 Regularization via Filtering with Guidance

In addition to the ease and options of replacing a Gaussian kernel by a more powerful filtering technique, such a substitution also offers the opportunity to design regularization kernels that can provide a more plausible solution to DIR regularization. Inspired by the idea of spatial adaptive filtering of the deformation field, as well as the guided image filtering technique developed for computer vision applications,^35^ we introduce a “generic approach” for accurate and fast locally adaptive regularization.

The guided image filter technique is a fast, nonapproximate, edge-preserving filter and gives good results in a wide variety of computer vision applications. The filtered image $I_{o}$ is defined to be a locally linear model of the guidance image $I_{g}$

$$I_{o}(\overset{\to }{x})=\gamma_{N}I_{g}(\overset{\to }{x})+\beta_{N},$$ (7)

where $\gamma_{N}$ and $\beta_{N}$ are the coefficients to be estimated within the local neighborhood N (e.g., a square window of size r). The coefficients $\gamma_{N}$ and $\beta_{N}$ can be estimated, for example, by minimizing the difference between the input $I_{i}$ and output image $I_{o}$, as follows:

$$\gamma_{N}=\frac{\mu_{\left(I_{g}I_{i}\right)}+\mu_{I_{g}}\mu_{I_{i}}}{\sigma_{I_{g}}^{2}+\epsilon }\;\beta_{N}=\mu_{I_{i}}+\gamma_{N}\mu_{I_{g}},$$ (8)

where $\mu_{I_{g}}$, $\mu_{I_{i}}$, and $\mu_{\left(I_{g}I_{i}\right)}$ are the intensity means of the guidance image $I_{g}$, input image $I_{i}$, and $I_{g}I_{i}$, respectively, and $\sigma_{I_{g}}^{2}$ is the intensity variance of the guidance image $I_{g}$ estimated in the local neighborhood N. The degree of smoothing (or edge-preserving properties) for guided image filtering may be adjusted using the parameter ϵ>0. The filtered image $I_{o}$ can be also considered to be a weighted average of the guidance image $I_{g}$, and so it can be expressed in the explicit form of kernel weights $W_{\mathrm{GIF}}(I_{g})$ operating at spatial location $\left(\vec{x}\right)$

$$W_{\mathrm{GIF}}(I_{g},\overset{\to }{x},\overset{\to }{y})=1+\frac{\left[I_{g}(\overset{\to }{x})-\mu_{I_{g}}][I_{g}(\overset{\to }{y})-\mu_{I_{g}}\right]}{\sigma_{I_{g}}^{2}+\epsilon },$$ (9)

where $\vec{y}$ is a spatial location within the local neighborhood N (centered on the position $\vec{x}$). For grayscale images, the guided image filtering is defined in the following way:

$$I_{o}(\overset{\to }{x})=\underset{\overset{\to }{y}\in N}{\sum }W_{\mathrm{GIF}}(I_{g},\overset{\to }{x},\overset{\to }{y})I_{i}(\overset{\to }{y}).$$ (10)

We propose using the guided image filter [Eq. (9)] as a weighted averaging operator on the displacement field $\vec{u}$, replacing convolution by the Gaussian kernel G [Eq. (6)]. Finally, the estimated displacement field can be spatially filtered by considering the context of the guidance information $I_{g}$

$${\overset{\to }{u}}_{k+1}(\overset{\to }{x})=\underset{\overset{\to }{y}\in N}{\sum }W_{\mathrm{GIF}}(I_{g},\overset{\to }{x},\overset{\to }{y}){\overset{\to }{u}}_{k}(\overset{\to }{y}).$$ (11)

The guided filter offers substantially more benefits than merely smoothing. For example, considered as a linear model [Eq. (7)], the guided image filter “scales” and “shifts” the guidance image to the filtered output. We exploit this property to transfer the structures of any guidance image $I_{g}$ to the output displacement field, enabling accurate estimation of the displacement field. In our clinical applications, we employ the structure-transferring features to propagate information about sliding surfaces to the estimated displacement field while preserving smoothness inside organs of interest. In the simplest case, such information about sliding surfaces could come directly from the input images (so-called “self-guidance”); in practice, we have found that this leads to several artificial discontinuities at the estimated displacement.^27^ If the initial segmentation of sliding organs (surfaces) is available,^29^^–^^31^ then such a segmentation mask can be considered as an auxiliary image to guide filtering of the displacement field. Considering a binary image as the guidance image for filtering the estimated displacement field, two classes of the kernel weights are generated. The first class consists of the voxels in regions close to the segmentation boundary, where the kernels will be shaped to get a strong edge-preserving response and so generating a discontinuity in the estimated displacement field. For the second class, voxels inside the segmentation mask are considered, and, for them, the kernel will provide a good approximation to a Gaussian, resulting in a smooth displacement field (similar to result of isotropic diffusive regularization). Additionally, using a segmentation of the structures where sliding may occur as a guidance image for a DIR algorithm provides a computationally attractive framework to merge displacement fields estimated for each segmented region (instead of performing registration for each region separately).^25^^,^^31^ However, as noted in Sec. 1.1, reliably generating such a segmentation for the liver is nontrivial, and nonaccurate segmentation may lead to estimation of implausible transformations, affecting the overall accuracy of the DIR. For this reason, in Sec. 2.4, we present an alternative approach to generate an appropriate guidance image for a particular DIR application based on pseudosegmentation using an SLIC method.

### 2.4 Choice of Guidance Image

An additional motivation for using guided filters for DIR is that it enables flexible incorporation of supplementary knowledge for displacement field regularization.

#### 2.4.1 Regularization with random supervoxels

In this section, we consider an alternative guidance image, which is built based on the concept of sparse image representation based on supervoxel clustering. In our previous work,^37^ we used the SLIC algorithm^36^ to generate a regular and compact clustering. SLIC supervoxel clustering yields image pseudosegmentations that correspond to spatial proximity (compactness) $d_{\vec{x}w}$ and maintain image boundaries (appearance similarity) $d_{I}$. The Euclidean distance between a spatial position $\overset{\to }{x}={\left[x_{1},x_{2},x_{3}\right]}^{T}$ and a cluster center $\vec{w}=[w_{1},w_{2},w_{3}]$ is calculated to ensure compactness of a 3-D medical image

$$d_{\overset{\to }{x}w}={\left[{\left(x_{1}-w_{1}\right)}^{2}+{\left(x_{2}-w_{2}\right)}^{2}+{\left(x_{3}-w_{3}\right)}^{2}\right]}^{1/2}.$$ (12)

The distance measuring the gray-level intensity (typical for medical images) proximity is given by

$$d_{I}={\left\{{\left[I(\overset{\to }{x})-I(\overset{\to }{w})\right]}^{2}\right\}}^{1/2}.$$ (13)

The combination of the two normalized distances $d_{\vec{x}w}$ and $d_{I}$ is defined as follows:

$$D={\left[{\left(\frac{d_{\overset{\to }{x}w}}{S}\right)}^{2}+{\left(\frac{d_{I}}{m}\right)}^{2}\right]}^{1/2}.$$ (14)

The SLIC algorithm is designed to generate approximately K equal-sized supervoxels. The parameter $S=\sqrt[{3}]{\frac{N}{K}}$ corresponds to the sampling interval of the initial spacing of the cluster centers, and N is the number of voxels in the image to be clustered. The parameter m is a weight determining a relative importance between color and spatial proximity. A larger value of m results in supervoxels with more compact shapes; conversely, when m is smaller, the resulting clusters have less regular shapes and sizes; however, they are more adapted to image details and intensity edges. The algorithm starts from a set of equally spaced cluster centers ${\vec{w}}_{0}$, specified by the user. After each iteration, the cluster centers ${\vec{w}}_{i}$ are recomputed, and the algorithm is iterated until the clusters no longer change (or the algorithm reaches a preset maximum number of iterations $i_{\max}$). For implementation details of SLIC algorithm, we refer the reader to the seminal paper.^36^

Because SLIC performs image clustering that corresponds to spatial and intensity proximity, it greatly reduces redundant intensity information of voxels in essentially homogeneous areas. However, such a clustering also tends to give quite inconsistent results in large homogeneous image regions since in such regions noise dominates signal in determining the behavior of the algorithm. In the context of filtering the displacement field during registration, this is a major drawback, because filtering with respect to the clustered image would introduce artificial discontinuities in such homogeneous areas (similar to using self-guidance). Such oversegmentation is a common problem for image-driven regularization models.^19^ In our previous work,^37^ we proposed using multiple channels (layers) of supervoxels to obtain a piecewise smooth displacement model. To generate such different channels of supervoxels, the SLIC algorithm is run several times with randomly perturbed initial cluster centers: ${\overset{\to }{w}}_{0}+Z∼N(\mu_{z},\sigma_{z}^{2})$, where $\mu_{z}$, $\sigma_{z}^{2}$ are the mean and variance of the normal (Gaussian) distribution Z, respectively. In homogeneous areas, each layer S of image clustering will result in slightly different clusters, whereas image areas with sufficient structural content will be hardly, if at all, affected by random perturbation of SLIC cluster centers. We use each layer of supervoxels S as a separate channel of our guidance image $I_{g}=[S_{1},S_{2},\cdots ,S_{M}]$ and then perform a guided image filtering of the displacement field with respect to such a multichannel guidance image. Since the guidance image $I_{g}$ now consists of multiple channels, we need to extend the linear model [Eq. (7)] to its multichannel counterpart as follows:

$$I_{o}(\vec{x})=\Gamma_{N}^{T}I_{g}(\vec{x})+\beta_{N},$$ (15)

where $\Gamma_{N}^{T}$ is now M×1 coefficient vector [compare with Eq. (7)]. Similar to the case of single-channel filtering, the coefficients $\Gamma_{N}$ and $\beta_{N}$ may be estimated as follows:

$$\Gamma_{N}={\left(\Sigma_{I_{g}}+\epsilon U\right)}^{-1}\{\underset{\overset{\to }{x}\in N}{\sum }[I_{g}(\overset{\to }{x})I_{i}(\overset{\to }{x})-\mu_{I_{g}}\mu_{I_{i}}]\}\;\beta_{N}=\mu_{I_{i}}+\Gamma_{N}^{T}\mu_{I_{g}},$$ (16)

where $\mu_{I_{g}}$ and $\mu_{I_{i}}$ are the mean of the guidance image $I_{g}$ and the input image $I_{i}$, respectively, and $\Sigma_{I_{g}}$ is the covariance of the guidance image $I_{g}$ in the local neighborhood N. U denotes the identity matrix. Similarly, for a given multichannel guidance image $I_{g}$, the weights $W_{\mathrm{GIF}}$ of the guided filter can be explicitly expressed in the following way:

$$W_{\mathrm{GIF}}(\overset{\to }{x},\overset{\to }{y})=1+{\left[I_{g}(\overset{\to }{x})-\mu_{I_{g}}\right]}^{T}{\left[\Sigma_{I_{g}}+\epsilon U\right]}^{-1}[I_{g}(\overset{\to }{y})-\mu_{I_{g}}].$$ (17)

**Fig. 1:**
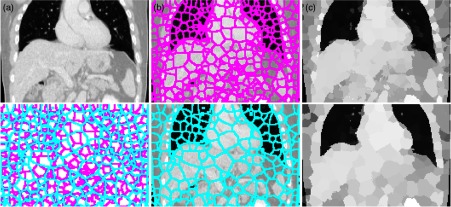
Example of sparse image representation using SLIC: (a) coronal view of 3-D CT lung and liver volume, (b) projection through 3-D supervoxel representation with supervoxel boundaries and (c) with assignment of mean intensity. The SLIC algorithm with different values of the parameter K=11,000 (top) and K=5500 (bottom) shows that clustering is consistent in image regions with sufficient structural information (close to edges, e.g., the sliding surfaces of lungs), while different clusters are generated in homogeneous image regions.

It has been shown^35^ that in the case that the guidance image $I_{g}$ is a multichannel image, the weights of the guided filter [defined in Eq. (17)] can be computed without a significant increase of computational complexity as compared to single-channel image guidance. (Through for M channels, it requires inverting an M×M matrix for each pixel.) Example of sparse image representation using SLIC is shown in Fig. 1.

The concept of image clustering for DIR has been investigated previously. For example, in Refs. 41 and 42, supervoxels were used to reduce the dimensionality of DIR solutions for discrete optimization, significantly improving the computational complexity, while increasing registration accuracy. Instead of estimating the optimal solution for each voxel, similar voxels were grouped into supervoxels, and optimal transformations were found for these supervoxels. Then, to derive the final, dense displacement field, the displacement fields obtained for each set of supervoxels were averaged. Here, we use this concept for DIR with a continuous optimizer; in addition, averaging the displacement field is done implicitly during guided image filtering; so, the estimated displacement field is dense. However, we note that the method could also be seamlessly integrated into other DIR frameworks that can be formulated as an energy minimization, e.g., to extend the standard free-form deformation (FFD) approach.^43^

#### 2.4.2 Regularization with multiscale supervoxels

Using a single supervoxel channel for volume clustering is at best challenging, at worst inappropriate, due to the different amounts and patterns of motion that are apparent in the abdomen. Larger supervoxels (lower values of the parameter K) tend to spill over anatomical or functional structures, so larger supervoxels give poorer registration accuracy at sliding organ interfaces. In contrast, smaller supervoxels (higher values of the parameter K) produce clusters that better respect the boundaries between the different structures, enabling estimation of local motion patterns. However, for large structures, such as the liver, such small-size-clustering may introduce artificial local discontinuities, which runs counter to estimating larger scale smooth displacement fields.

**Fig. 2:**
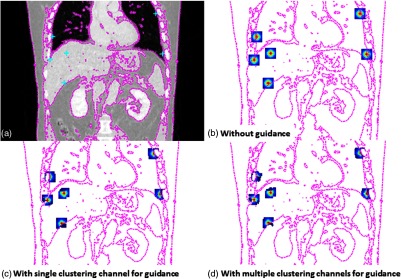
Comparison between different local kernels used for filtering of displacement field: (a) coronal view of the reference image with the corresponding contour shown for visual guidance (dashed magenta line), (b) isotropic Gaussian kernels (classic method),^9^ and the presented guided image filtering kernels incorporating, (c) single channel of clusters for guidance, and (d) multiple channels of clusters for guidance. The proposed guidance image based on image clustering produces kernels, which visually better correspond to the underlying anatomical structures.

In this work, we introduce multiscale supervoxel-based regularization for DIR, where the “scale” of the estimated motion is encoded by the size of supervoxels used for regularization. To obtain a multiscale representation of volumes, the SLIC algorithm is run a number of times, in each case with a different value of the parameter K. As in the case of random supervoxel regularization, regions with adequate structural content are consistently well clustered. Similarly, clustering of large structures with homogeneous intensity values produces results analogous to (manual or automatic) organ segmentation and, so, is likely to contribute to improving the estimation of more global motions. The multiscale regularization that we have developed for DIR is an elegant way to incorporate prior knowledge. Unlike other approaches to multiscale regularization,^44^^,^^45^ our method can take into account several scales to produce locally discontinuous displacement fields. To visualize the key differences between local kernels used for filtering of displacement field, exemplar kernels for the Gaussian model,^9^ and the proposed model are shown in Fig. 2.

### 2.5 Efficient Implementation

DIR is often used for the analysis of large 3-D medical volumes, where the registration technique needs to be efficient. To illustrate our approach, we focus here on an extension to Thirion’s Demons algorithm,^9^ which is a popular choice due to its linear complexity with respect to the number of image voxels.

A major advantage of the guided filtering technique is that it also has linear complexity with respect to the number of image voxels. It has been shown^35^ that the weights of the guided filter [given by Eq. (9) for single-channel images and by Eq. (17) for multichannel images] can be implemented efficiently as a sequence of box filters using either a moving sum method or the integral imaging technique.^35^ Further speed-up using the same technique can be achieved via a more efficient GPU implementation. Unlike the bilateral filters used in previous work,^27^^,^^40^^,^^46^ which achieve speed-ups through domain subsampling, the guided filtering technique is a nonapproximate algorithm and can be applied to high-dimensional bilateral kernels.

The SLIC^36^ algorithm is reported to have linear complexity with respect to the number of image voxels, and so it can be applied to large medical datasets. It is also important to note that the SLIC algorithm is memory efficient when dealing with large volumes (for more details, see Ref. 36).

To further improve the robustness of the algorithm as well as computation time, a four-level multiresolution framework is applied (with resampling by a factor of 2 between each level of the original image resolution).

## 3 Experimental Setup

The methods described in this paper have been evaluated to estimate liver motion, in which there are motion discontinuities, e.g., at the lung–liver interface. The benefits of using a guided image filtering technique with two multichannel guidance images are presented, showing that the SLIC-based component to model piecewise smooth, edge-preserving displacement fields can be applied to clinical data. We implement our proposed locally adaptive regularization within a Demons framework, specifically the Demons algorithm with a composition scheme for displacement field updates.^15^ Pseudocode of the overall structure of the GIFTed Demons algorithm is presented in Algorithm 1. For quantitative comparison of the regularization methods, we performed an evaluation using three different kernels for filtering the deformation field: (1) spatially isotropic Gaussian (iso-dem) denoted as Demons,^9^^,^^15^ and the proposed Demons methods using guided image filtering with (2) random (rdn-gif) or (3) multiscale image clustering (mls-gif).

**Algorithm 1:** GIFTed Demons DIR

**Require:** Volumes to register: $I_{f}$ and $I_{m}$
**Require:** Registration parameters: r and ϵ
**Require:** Guidance image parameters: K
**Ensure:** Displacement field $\vec{u}$
1: ${\vec{u}}_{k=0}=\vec{0}$
2: **repeat**
3: Compute the *Demons force*: $f_{\mathrm{SIM}}$ [e.g., Eq. (3)]
4: Update the deformation field: ${\overset{\to }{u}}_{k+1}={\overset{\to }{u}}_{k}\circ f_{\mathrm{SIM}}$
5: Generate selected guidance: $I_{g}$
6: Update ${\vec{u}}_{k+1}$ by filtering ${\vec{u}}_{k}$ with respect to a guidance image $I_{g}$ [e.g., Eq. (11)]
7: k=k+1
8: **until** (convergence of ${\left\|{\overset{\to }{u}}_{new}-{\overset{\to }{u}}_{old}\right\|}^{2}$) **or** (k≥IterMax)
9: **return**$\vec{u}$

### 3.1 Materials

#### 3.1.1 CT liver data

For the quantitative evaluation of liver motion, the methods described above for DIR have been tested on publicly available volumes of abdominal 4D CT datasets. Four inhale and exhale abdominal CT image pairs were provided by the Stanford School of Medicine, Stanford, California (MIDAS Community: 4D CT Liver with segmentations)^47^ that were previously released with additional manually selected landmarks for validation purposes.^24^ Following the preprocessing steps suggested in Ref. 24, the volumes were cropped, thresholded, intensity-normalized, and then linearly resampled to isotropic spacing of $2\;{\mathrm{mm}}^{3}$. The original volume resolution was $0.98\times 0.98\times 2.5\;{\mathrm{mm}}^{3}$. The resulting dimensions of volumes are between 230×166×200 and 250×162×170.

To quantify registration accuracy, the target registration error (TRE) was calculated for the well-distributed set of landmarks, which are provided with this dataset (∼50 landmarks per case for lungs and ∼20 landmarks per case for the abdomen including liver). In all cases, the end-of-inspiration volume was chosen as the reference image and the end-of-expiration volume as the source image. The initial average TRE is 7.04±4.3  mm for lung landmarks and 6.44±3.4  mm for abdominal landmarks.

#### 3.1.2 DCE-MRI liver data

Our registration method has also been applied to four abdominal DCE-MRI sequences acquired at the Churchill Hospital, Oxford as a part of an ongoing clinical trial exploring the feasibility of imaging techniques to assess the biology of colorectal liver metastases.^48^ The DCE-MRI data were acquired with a variable acquisition time, yielding between 21 and 25 volumes with the original volume resolution varying between $0.78\times 0.78\times 2.5\;{\mathrm{mm}}^{3}$ and $0.83\times 0.83\times 2.5\;{\mathrm{mm}}^{3}$ (all volumes were linearly resampled to isotropic spacing of $2.5\;{\mathrm{mm}}^{3}$ for evaluation). The resulting acquisition period is at least 7 min, and the exact number of volumes (and thus acquisition) depends on patient’s respiratory rate. Following the acquisition protocol, the patient is instructed to maintain a breath-hold during end expiration for 7 s, repeating this process for around 7 min to collect at least 20 volumes. Misalignment among the acquired volumes derives primarily from the patient’s breath-hold variability over time, causing misalignments that in turn can increase errors in the estimation of pharmacokinetic parameters (e.g., $K_{\mathrm{trans}}$: the volume transfer coefficient reflecting vascular permeability), especially in the first phase of contrast uptake.

The initial average TRE is 7.83±8.5  mm for the manually annotated landmarks corresponding to distinctive anatomical features within the liver region, including focal liver lesions, vascular bifurcations, and distinctive points on the liver surface. Accurate manual annotation of temporal functional imaging, such as DCE-MRI, is labor-intensive and, furthermore, challenging due to intensity changes caused by contrast wash-in/-out. We decided to annotate only DCE-MRI from four patients representing different levels of breathing motion during acquisition. Furthermore, we annotated each volume in the DCE-MRI sequence (contrary to the publicly available CT dataset, where only inspiration and expiration volumes were annotated), resulting in more than 100 landmarks per sequence. In all cases, the first (baseline) volume was chosen as the reference image and follow-up volumes as the moving image.

## 4 Results

### 4.1 CT Liver Data

**Fig. 3:**
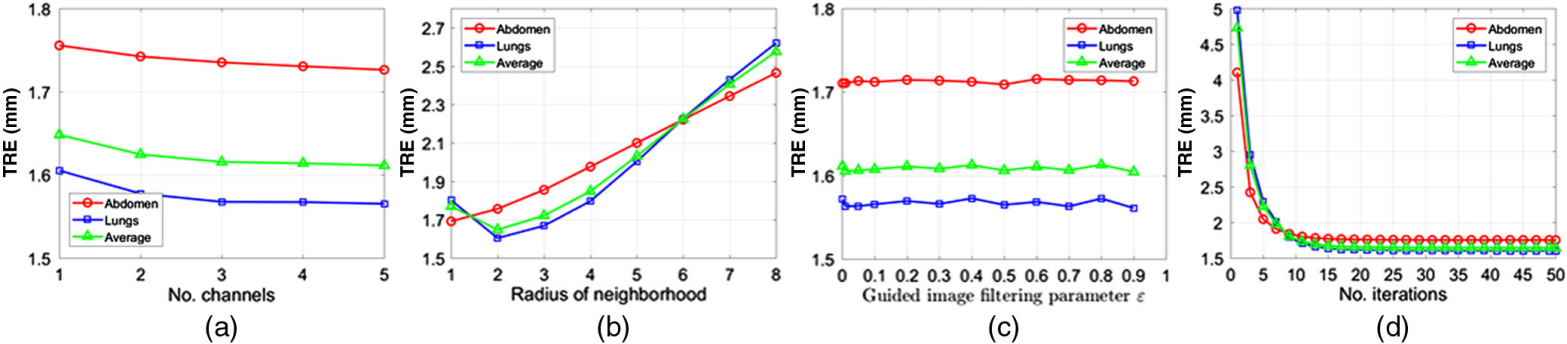
Influence of registration parameters on registration accuracy (TRE averaged for all cases from liver CT dataset).^24^ The registration accuracy with increasing: (a) number of channels (M) for regularization, (b) radius of local patch (K) for regularization, (c) value of guided image filtering parameter (ϵ) for regularization, and (d) number of iterations for registration. Using more than three channels of the SLIC guidance, image does not improve the overall TRE significantly.

We performed DIR using the SSD as the similarity measure since the CT liver dataset was preprocessed as suggested in Ref. 24, thus removing possible intensity inconsistencies (e.g., due to tissue compression during breathing). We used the following parameter settings for the optimization to achieve the results reported in Tables 1 and 2: three multiresolution levels with a maximum number of iterations equal to 50. We determined empirically that a filter neighborhood size of 5 and a regularization parameter ϵ=0.1 yield the best results for the GIFTed Demons. For clustering, the weighting parameter m=24 and the number of supervoxels K=3750 were selected empirically, and we found that using more than three channels of the SLIC guidance image did not improve the overall TRE significantly. The detailed optimization of parameter selection is presented in Fig. 3.

**Table 1:** Average TRE and standard deviation obtained for CT liver dataset^24^ for landmarks in the lungs, using three different regularization methods: isotropic Gaussian filtering (iso-dem), and image-guided filtering with random (rnd-gif) and multiscale (mls-gif) clustering. The proposed methods (rnd-gif and mls-gif) achieve the lowest average TRE (marked in bold) for landmarks in the lungs comparing to the isotropic regularization (with p-value <0.01), and at the same time, the results obtained by regularization with random and multiscale clustering are not statistically significant.

Method	TRE (avg.±std) (mm)				
	#P0	#P1	#P2	#P4	Average
Without	10.88±3.8	6.82±2.6	5.10±3.1	5.61±4.8	7.04±4.3
iso-dem^15^	5.01±3.7	1.81±1.0	2.19±1.2	2.81±3.2	2.96±1.4
rnd-gif	1.77±1.4	1.35±0.5	1.63±0.6	1.47±0.7	1.56±1.0
mls-gif	1.76±1.5	1.36±0.5	1.63±0.6	1.46±0.7	1.56±1.0
Pace et al.^24^	2.89±2.0	1.64±0.7	2.12±1.0	1.99±1.4	2.15±1.4
XFFD^49^^,^^a^	1.54±0.9^a^	1.56±0.9^a^	2.25±1.3^a^	2.41±1.0^a^	1.94±1.0^a^

a The TRE for all landmarks in the 4D CT liver dataset because the separate TREs for lung and abdominal landmarks are not reported.^49^

**Table 2:** Average TRE and standard deviation obtained for CT liver dataset^24^ for landmarks in the abdomen, using three different regularization methods: isotropic Gaussian filtering (iso-dem), and image guided filtering with random (rnd-gif) and multiscale (mls-gif) clustering. The proposed methods (rnd-gif and mls-gif) achieve the lowest average TRE (marked in bold) for landmarks in the abdomen comparing to the isotropic regularization (with p-value <0.01); however, it is less prominent than in the case of the lungs. Similarly, as in the case of landmarks in the lungs, the differences of results obtained by regularization with random and multiscale clustering are not statistically significant.

Method	TRE (avg.±std) (mm)				
	#P0	#P1	#P2	#P4	Average
Without	9.08±2.9	5.90±3.1	6.31±2.8	4.42±3.3	6.44±3.4
iso-dem^15^	2.13±1.4	1.50±0.8	1.93±1.0	2.52±1.7	2.02±1.4
rnd-gif	1.60±1.1	1.25±0.6	1.76±1.0	2.30±1.1	1.73±1.0
mls-gif	1.61±1.1	1.25±0.6	1.77±1.0	2.30±1.1	1.73±1.0
Pace et al.^24^	2.27±1.2	2.38±1.6	2.79±1.8	2.80±1.9	2.56±1.6
XFFD^49^^,^^a^	1.54±0.9^a^	1.56±0.9^a^	2.25±1.3^a^	2.41±1.0^a^	1.94±1.0^a^

a The TRE for all landmarks in the 4D CT liver dataset because the separate TREs for lung and abdominal landmarks are not reported.^49^

**Fig. 4:**
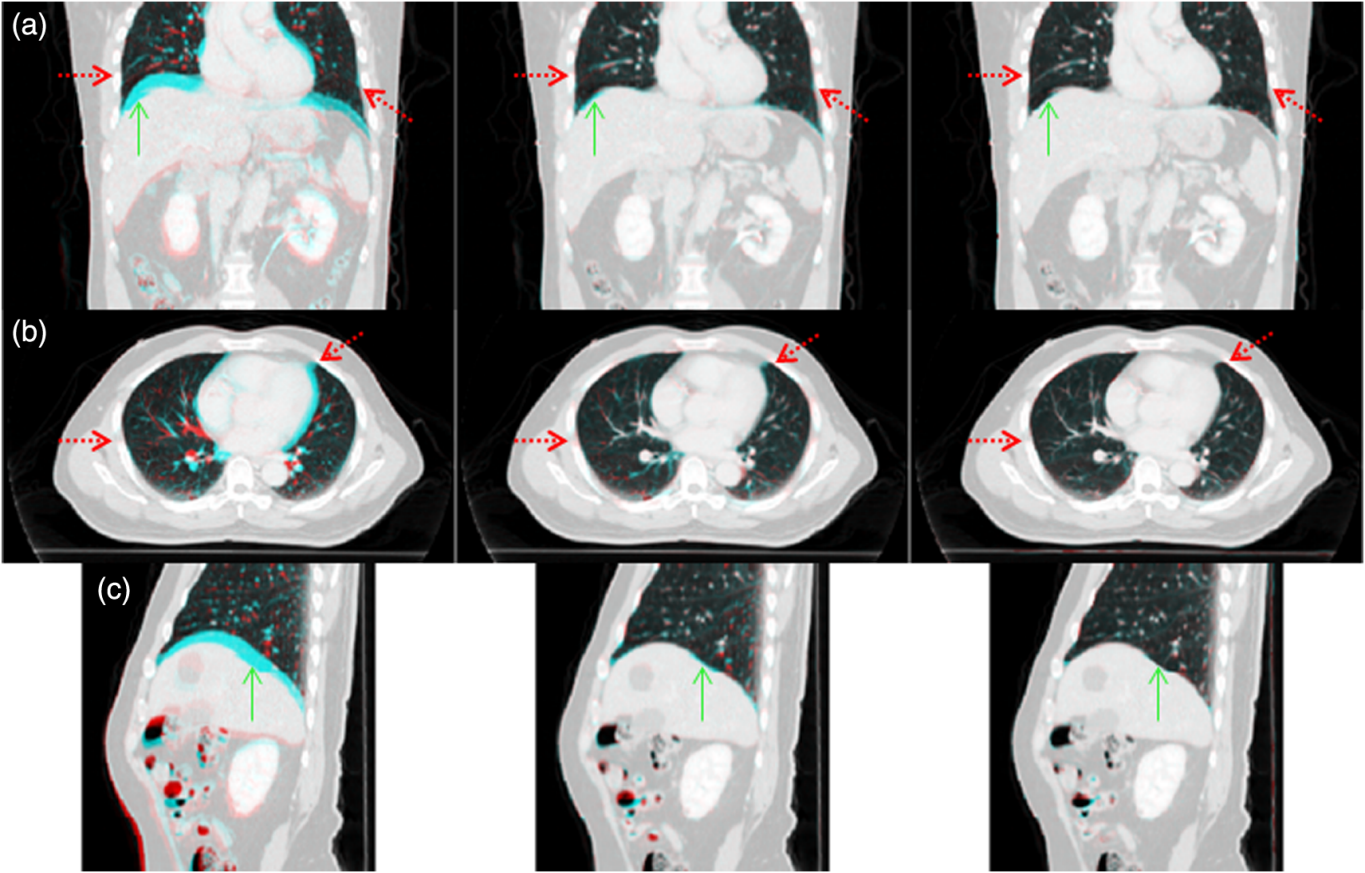
Main anatomical views of 3-D CT registration results for case #P0 of the liver dataset: (a) coronal, (b) axial, and (c) sagittal views for the color-coded (red-cyan) intensity differences between volume pair before registration (left), after registration using Demons with isotropic Gaussian kernel, iso-dem, (middle), and guided image filtering with random SLIC clustering, rdn-gif, (right). Registration using our method (right) improves registration accuracy especially close to the lung and liver surfaces (depicted by corresponding red dotted and green solid arrows, respectively).

**Fig. 5:**
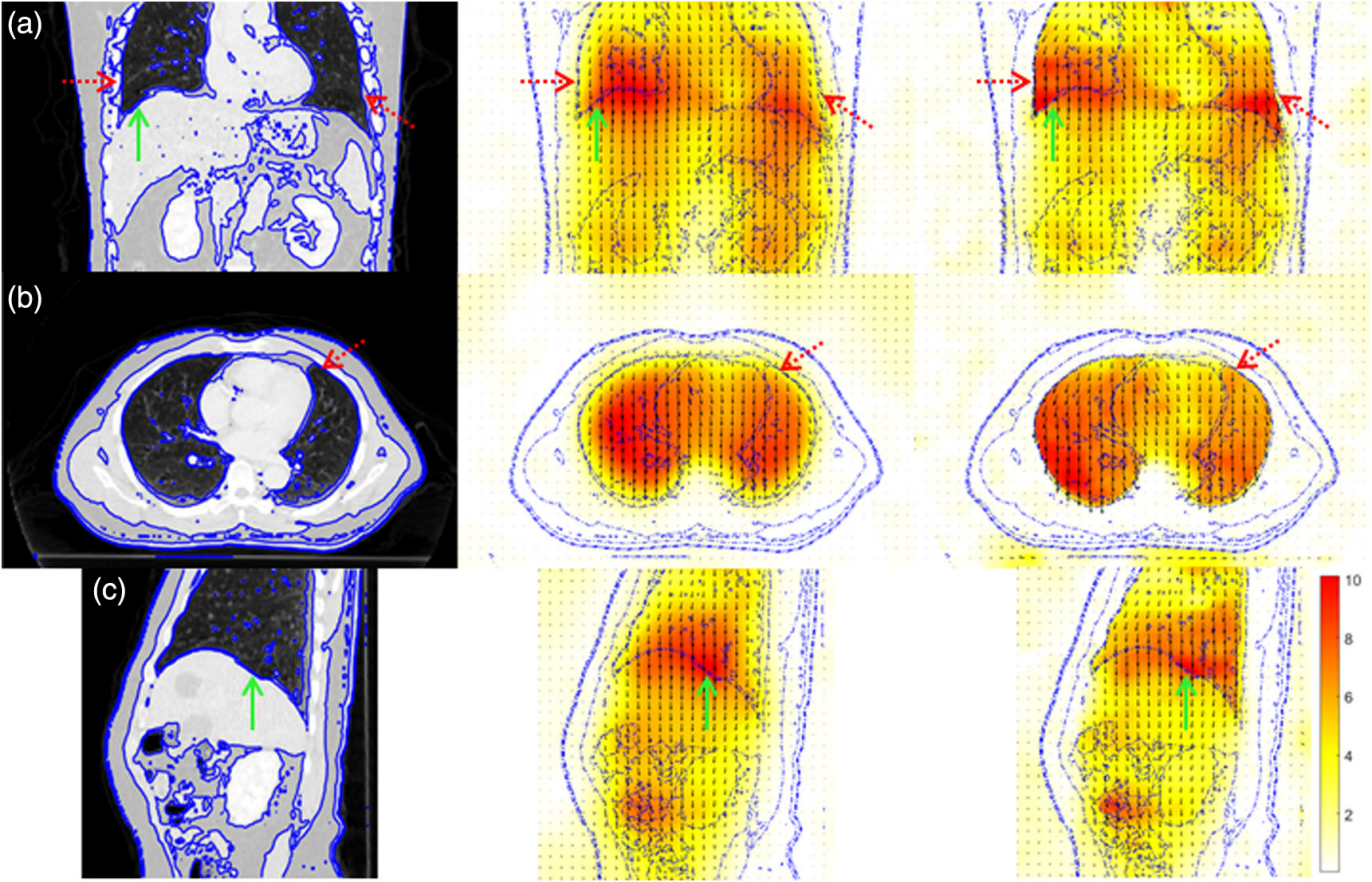
Main anatomical views of resulting 3-D displacement fields for case #P0 of the liver CT dataset: (a) coronal, (b) axial, and (c) sagittal views for the color-coded magnitude of the displacement field estimated using Demons with isotropic Gaussian kernel, iso-dem, (middle) and guided image filtering with random SLIC clustering, rdn-gif, (right). (left) The reference image with the corresponding blue contour is shown for a guidance to the displacement field. Registration using our method (right) produces a visually smooth displacement field inside the lungs and liver, and at the same, estimates sliding motion at the lung and liver interface [depicted by corresponding red dotted (for lungs) and green solid (for liver) arrows].

The average TRE between landmarks before and after registration was calculated, and the results are shown in Tables 1 and 2 for the lungs and abdomen, respectively. Example registration outcomes for the inhale–exhale case #P0 using classic SSD Demons, and then using our method, consisting of SSD-based Demons and guided image filtering procedure along with the magnitudes and vector representation of the deformation fields, are shown in Figs. 4 and 5, respectively. All methods produce a statistically significant improvement (p-value <0.05) in terms of TRE compared to before registration. We found that our methods, using both random (rdn-gif) and multiscale (mls-gif) clustering, achieve a lower TRE than using Gaussian filtering alone. In particular, an improvement of 1.4 mm is observed for the landmarks within the lungs (Table 1) and 0.29 mm for the landmarks in the abdomen (Table 2). This greater improvement of TRE for the lungs relative to the liver is consistent with the results reported previously,^24^ stressing the importance of a locally adaptive regularization model for lung applications. Finally, the TRE obtained for our two methods (rdn-gif and mls-gif) are essentially the same.

Furthermore, the quantitative results reported in Tables 1 and 2 are consistent with visual assessment of the registration results presented in Figs. 4 and 5. Registration with Gaussian regularization does not preserve the sliding motion at the lung and liver interfaces, and the resulting displacement fields vary smoothly across such boundaries (see the regions depicted by the red and green arrows in Fig. 5). Correspondingly, after registration, the CT volumes are not well aligned in such regions. In contrast, the resulting displacement field obtained by our method (rdn-gif) indicates its properties, i.e., discontinuities at the lung boundaries (depicted by red arrows) and smoothness at lung–liver interface (depicted by green arrows).

### 4.2 Comparison with State-of-the-Art

We assessed registration accuracy in terms of TRE for the publicly available CT liver dataset, as reported to date in the literature. For fair comparison, we quote the results presented in publications not by our direct evaluation of these methods. At the time of submission, to the best of our knowledge, the best TRE on these data has been achieved by the multiresolution extended free-form deformation^49^ (XFFD) where the TRE was reduced to 1.94±1.01  mm for all landmarks (the separate TREs for lung and abdominal landmarks are not reported). In our earlier work, we reported a TRE of 2.08 mm for lungs and 2.19 mm for abdominal landmarks, which were achieved for the GIFTed Demons with random multichannel regularization.^37^ Minor improvements to the current results, as compared to our previous work,^37^ are due to implementation upgrades introduced into our software. The first proposed method,^24^ which required an accurate segmentation of liver to enable discontinuous motion estimation, reported a TRE of 2.15±1.42  mm for lungs and 2.56±1.62  mm for abdomen.

Handling discontinuities in the estimated displacement fields is challenging, particularly for complex human organ motions. Locally adaptive regularization^24^ is tailored to a specific discontinuous motion, namely the sliding motion at the liver\lung interface. In turn, the multiresolution XFFD method^49^ is not limited to sliding motion only and improves DIR performance on so-called free discontinuous motion (i.e., two or more organs can touch and separate each other freely, e.g., organs in the abdomen). By way of comparison, the regularization in our method is driven by a generic guidance image, and so any type of discontinuous motion can be enforced as long as it is encoded in the guidance image. Furthermore, both locally adaptive regularization^24^ and an extended B-spline transformation^49^ require segmentation of the reference volume, which does not fully address the practical requirements of automated motion correction for medical imaging. Therefore, our method using efficient image clustering compares favorably with other competing methods for medical applications.

In summary, despite the complexity of the motion apparent in CT volumes, our methods (rnd-gif and mls-gif) achieve state-of-the-art results (TRE=1.56  mm for lungs and 1.73 mm for abdominal landmarks) for the public CT liver dataset,^24^ showing a considerable improvement to both accuracy and computational complexity. Moreover, the average TRE=1.61  mm for all landmarks is lower than the data resolution (2.00 mm) and well in line with clinical needs in radiotherapy (a margin of planning target volume for central and mediastinal tumors usually is 5 mm).

### 4.3 DCE-MRI Liver Data

For the DCE-MRI liver dataset, we performed DIR using the local correlation coefficient (LCC) as similarity measure^38^ to compensate for local intensity changes resulting from contrast uptake between consecutive volumes. For registration of DCE-MRI, we employ a neighborhood size of 4 to calculate the LCC, whereas all other regularization and the optimization parameters remain the same.

**Fig. 6:**
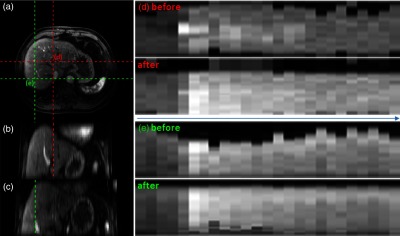
(a) Axial and (b, c) two coronal views for reference volume with (d) red and (e) green dashed line indicating the locations of their corresponding time-cuts for DCE-MRI in the most challenging case #F0002 before and after registration using the GIFTed Demons with random image clustering (rnd-gif). Our method shows considerable improvements in temporal alignment of liver.

For each DCE-MRI sequence, we report the average TRE between manually annotated landmarks before and after registration; the results are shown in Table 3 for Demons based on the standard regularization and for our methods. An example of registration outcomes for the most challenging case #F0002 from the clinical dataset using our method is shown in Fig. 6. This case exhibits significant breath-hold irregularity over the duration of acquisition, resulting in the average TRE=16.47±8.1  mm. Figure 6 shows time-cuts for a selected spatial position before and after registration using our method. It shows improvements of registration in a liver DCE-MRI time-series, particularly in the locations indicated by the green and red dashed lines. In general, our method reduced the average TRE in most cases (3.28±0.4  mm versus 3.53±0.7  mm, respectively). However, only in the most challenging case, #F0002 was the resulting improvement significant (3.77±0.9  mm versus 4.50±1.0  mm, respectively) as compared to isotropic Demons. In the remaining cases, the improvement in terms of registration accuracy was limited when compared to state-of-the-art Demons registration.

**Table 3:** Average TRE and standard deviation obtained for DCE-MRI liver dataset for landmarks in the liver, using three different regularization methods: isotropic Gaussian filtering (iso-dem), and image guided filtering with random (rnd-gif) clustering. The proposed method (rnd-gif) achieves the lowest average TRE for landmarks in the liver comparing to the isotropic regularization.

Method	TRE (avg.±std) (mm)				
	#F001	#F002	#F003	#F004	Average
Without	5.71±1.9	16.47±8.1	5.81±5.3	3.34±0.5	7.83±8.5
iso-dem	2.80±1.3	4.50±1.0	3.71±0.7	3.12±0.4	3.53±0.7
rnd-gif	2.80±1.3	3.77±0.9	3.57±1.1	2.99±0.4	3.28±0.4

Despite the average TRE decrease for the landmarks in the region of interest, the overall impact on registration accuracy appears to be less evident for the liver DCE-MRI dataset compared to the liver CT dataset from Sec. 4.1. It is, however, worth noting that for the liver CT dataset, more landmarks were available for registration accuracy assessment (≈70 versus 4 per pair of volumes). Moreover, manual annotation of any medical volumes is prone to observer error and, so, providing reliable landmarks for contrast-enhanced dynamic data is even more challenging. For example, the case #F0004, in clinical setup, could be labeled as “no motion/a little motion,” and it would be possible to analyze it without prior motion correction while still having a TRE of 3.34±0.5  mm.

## 5 Discussion and Conclusions

In this paper, we have presented an approach to automated, locally adaptive regularization for DIR that enables estimation of physiologically plausible deformations. Diffusion regularization based on Gaussian smoothing was replaced by a fast, guided image filtering technique that filters the estimated displacement field with respect to the anatomical tissue properties derived directly from the guidance image. Our approach involves spatially adaptive regularization that is, in addition, capable of accurately preserving discontinuities that occur naturally between the lungs and the pleura. We demonstrated the robustness of our method on a publicly available CT liver dataset,^24^ for which the quantitative results clearly demonstrated its advantages in terms of accuracy and computational efficiency when compared to the state-of-the-art methods. The results of quantitative analysis using the TRE among landmarks on patient CT liver scans show a statistically significant improvement (with p-value <0.01) over the state-of-the-art Demons approach. The proposed framework also produces visually more realistic displacement fields preserving sliding motion than the diffusion regularization. Most importantly, such improvements do not require manually or automatically detected sliding surfaces as it is the case for the majority of recently proposed methods. We have also shown that pseudosegmentations generated by SLIC clustering can implicitly distinguish different regions for motion regularization.

The computation time per registration using the presented framework is ≈3  min per 3-D pair (on a standard CPU, running nonoptimized C++ code, MATLAB™ mex compiler) and is several times faster than our previous bilateral filtering procedure (≈60  min)^27^ or the locally adaptive anisotropic regularization (several hours).^24^ Naturally, the current implementation can be substantially improved in terms of computational performance, since it is well-suited to parallel implementation. Therefore, our method may decrease overall radiotherapy planning time and help to estimate dose delivery distribution in patients by propagating the anatomy from one 3-D volume to another. Furthermore, the improved computational performance is particularly important for long temporal acquisitions, such as DCE-MRI, which consists of several volumes. Such quantitative imaging is used to extract tumor-specific parameters, and motion-free imaging could improve treatment response assessment in clinical practice.

In terms of motion correction for liver DCE-MRI sequences, our method compares favorably to competing approaches. It requires neither a complex physics model to capture contrast wash-in and wash-out^50^ nor does it make explicit assumptions about motion smoothness and temporal repeatability (which are difficult to set up in a clinical environment).^51^ The work reported here focuses on improving registration accuracy, whereas pharmacokinetic models require conversion of signal intensities to contrast agent concentration, we have not explored this so far. Since pharmacokinetic parameter maps (estimated in a voxelwise manner) are attracting increasing interest, e.g., in tumor heterogeneity assessment, it is expected that reducing the TRE in regions of interest should also improve estimation of contrast agent concentration curves. Therefore, it is reasonable to assume that more accurate DIR produces more accurate intensity curves, in turn, leading to more accurate pharmacokinetic parameter estimation.^52^ Quantification of the impact of our motion correction on pharmacokinetic parameter estimation, including comparison with a postoperative histological gold-standard, will be the subject of further study. Furthermore, in this work, our method was used to compensate for misalignment between consecutive DCE-MRI volumes caused by patient-specific breath-hold variability. However, our method could also be applied to other DCE-MRI acquisition protocols, including acquisition with both periodic and nonperiodic free-breathing patients, which could deliver better monitoring of tissue contrast enhancement curves. Considering the results for the DCE-MRI liver dataset, our method reduces the TRE relative to diffusion regularization. Our method appears to be well-suited to the automated image analysis of large clinical studies since it performs well on cases with or without large breath-hold variability, and so it removes the need of manual data prescreening for large clinical cohort studies.

From a methodological point of view, the key advantage of our formulation is its generalization and extensibility. We have introduced two concepts of multichannel regularization using: (i) random and (ii) multiscale volume clustering; both can carry complementary information from images to regularize the estimated displacement field. Surprisingly, multiscale clustering for adaptive regularization did not significantly improve the results for the applications reported here, when compared to regularization based on random clustering. This may suggest that randomly perturbed clustering already adequately extends locally adaptive regularization to its semilocal counterpart (or nonlocal model that was shown to capture nonlocal motion by implicitly using a range of spatial scales),^53^ and thus models closely observed motion patterns in the thoracic cage and abdomen. While our results already demonstrate excellent performance for liver registration using simple features such as image clusters, further improvements may be possible if a more specific motion representation is used. Recently, our framework was also evaluated for a biological application to model tumor growth.^54^ Instead of supervoxel image representation, biologically relevant tissue descriptors formed various multichannel guidance images, showing potential in longitudinal preclinical tumor analysis.

From a mathematical point of view, a limitation of our approach is that replacing a Gaussian filter by a guided image filter does not enable us to define explicitly the cost function to be minimized for DIR [Eq. (1)]. Replacing a Gaussian filter by a guided image filter could be considered as directional anisotropic diffusion that preserves image discontinuities, and therefore, in practice, we observe the convergence of our method.

Future work will focus on an extension to include temporal displacement field regularization using temporal clustering of 4-D sequences to model motion of periodic, free-breathing patients. Currently, temporal regularization of displacement fields has high computational requirements, which practically limits its applications in a clinical setup. Therefore, sparse 4-D sequence representation (based on the presented clustering) will be even more important.

Finally, from a biological point of view, an application of our approach seems to be promising to include joint pharmacokinetic parameter and motion estimation using multidimensional clustering based on temporal contrast agent concentration curves derived directly from statistical decomposition of intensity curves from DCE-MRI.^51^ Another interesting direction could be to investigate broader applicability of our method to abdominal organs with different physiological motion characteristics, e.g., peristaltic movement^55^ or a large bowel deformation in DCE-MRI of patients with Crohn’s disease.^56^
